# Human Flourishing in Cross Cultural Settings. Evidence From the United States, China, Sri Lanka, Cambodia, and Mexico

**DOI:** 10.3389/fpsyg.2019.01269

**Published:** 2019-05-29

**Authors:** Dorota Wȩziak-Białowolska, Eileen McNeely, Tyler J. VanderWeele

**Affiliations:** ^1^Sustainability and Health Initiative (SHINE), Department of Environmental Health, Harvard T.H. Chan School of Public Health, Boston, MA, United States; ^2^Human Flourishing Program, Institute for Quantitative Social Science, Harvard University, Cambridge, MA, United States; ^3^Department of Epidemiology, Harvard T.H. Chan School of Public Health, Boston, MA, United States

**Keywords:** Flourish Index, human flourishing, Secure Flourish Index, Cambodia, Sri Lanka, Mexico, China, United States

## Abstract

This paper investigates human flourishing in five culturally distinct populations. Empirical differences in human flourishing were examined using the recently proposed Flourish Index (FI) and Secure Flourish Index (SFI). Five domains for human flourishing are proposed for FI: (D1) happiness and life satisfaction; (D2) physical and mental health; (D3) meaning and purpose; (D4) character and virtue; and (D5) close social relationships. Specification of SFI was augmented by an additional financial and material stability domain (D6). Psychometric properties of FI and SFI were examined using data from the SHINE Well-Being Survey. Between June 2017 and March 2018, a total of 8,873 respondents participated in the study – in the US (4083 participants), Sri Lanka (1284 participants), Cambodia (587 participants), China (419 participants), and Mexico (2500 participants). US participants were customers of a financial institution, while non-US participants were clothing industry workers in the supply chain of a global brand. Exploratory and confirmatory factor models were used to validate the proposed indices. An exploratory approach informed analysis for item groupings. Confirmatory factor models were used to investigate the hierarchical structure of the indices. Configural, metric, and partial scalar measurement invariance were established, which not only supported the universal character of the indices but also validated use of the indices for culturally distinct populations. Findings from our study enrich our knowledge about human flourishing in five culturally distinct populations. With the exception of happiness and life satisfaction, respondents in the US, despite enjoying the highest financial and material stability, scored the lowest in all other domains of human flourishing. Respondents in China excelled in close social relationship and health domains. In addition to life satisfaction and happiness, character and virtue were relatively high in Cambodia. Respondents in Mexico, despite having the lowest scores in financial and material stability, had the greatest meaning and purpose to their lives. Respondents in Sri Lanka were the least happy and satisfied with life.

## Introduction

In this study we present two measures of human flourishing with psychometric support for their application to culturally distinct populations. Comparisons were made between states of human flourishing in Cambodia, China, Mexico, Sri Lanka, and United States. These measures were proposed by VanderWeele (b) in previous commentary. Although both measures have been shown to have satisfactory psychometic properties in the working adults population in the US (Weziak-Bialowolska et al., 2019), they had never been tested empirically in the culturally distinct populattions, which, due to differences in cultural norms about ideal emotional states, may be source of systematic variations in reported levels of well-being (National Research Council, 2013). This paper fills this gap.

VanderWeele (2017a,b) argues that human flourishing – derived from Latin *florere* (to blossom) – extends beyond psychological well-being and might be understood as a state in which all aspects of a human life are good. He proposed five domains of human flourishing corresponding to important aspects of human life, which, if satisfactory, would constitute a state of human well-being. These are: D1. happiness and life satisfaction; D2. physical and mental health; D3. meaning and purpose; D4. character and virtue; and D5. close social relationships. While D1, D3, and D5 are usually included in measures of social well-being, eudemonic or hedonic happiness (see for example: Ryff, 1995; Ryff and Keyes, 1995; Keyes, 1998; Diener et al., 2010; Huppert and So, 2013; Su et al., 2014), the remaining two are usually ignored. With respect to the physical and mental health domain, it may be argued that measures of mental state are covered by composite measures of psychological well-being. There is, however, seeming agreement that flourishing transcends mental health. Additionally, although physical health is central to personal well-being, it is not referenced by the construct for psychological well-being.

If the intention is to assess complete human well-being or human flourishing, qualities beyond the psychological should be taken into account. Such qualities not only include physical and mental health but also cover character and virtue. Physical health, and, to a lesser extent, character and virtue, are almost completely ignored by the psychological literature, while in philosophical literature their importance to human flourishing is established (Pieper, 1966; Aristotle, 2009; Alexandrova, 2013; Baril, 2016).

As suggested by VanderWeele (2017b), an understanding of how humans flourish should extend beyond the momentary state. Therefore, an additional domain – D6. material and financial stability – was proposed to allow evaluation of the sustainability of a flourishing state.

Since no prior investigation of the validity or reliability of the FI and SFI – as proposed by VanderWeele (2017b) – has yet been undertaken in culturally distinct populations, this study examines the psychometric properties of the FI and the SFI and provides empirical evidence of validity and reliability. First, exploratory factor analysis (EFA) was used to investigate whether the grouping of items into domains of flourishing – as suggested by VanderWeele (2017b) – was justified by the data. Second, confirmatory factor analysis models were used to investigate how the complex structure of the indices (items grouped into domains, domains grouped into the indices) was reflected in the data. Third, reliability of both indices was examined. Finally, measurement invariance properties (Byrne et al., 1989; Meredith, 1993; Byrne and van de Vijver, 2010; Wȩziak-Białowolska, 2015; Putnick and Bornstein, 2016) of the FI and the SFI were assessed for evidence of cross-cultural universality.

This study also contributes to a global understanding of human flourishing by examining human flourishing domains in populations of garment workers in a well-known brand’s supply chain in Cambodia, China, Mexico, Sri Lanka and in the population of customers of a financial institution in the United States. In other words, the study was conducted in three populations with interdependent cultural norms (China, Cambodia, and Sri Lanka), one population with independent cultural norms (US) and one population which is perceived as a mixture of interdependent and independent cultures (Mexico). This was considered particularly relevant, since research had pointed to the influence of cultural elements on levels of well-being, purpose, and meaning in life and happiness (Steger et al., 2008; Kitayama et al., 2010) and on perceptions of “right” and “wrong” and the moral decision making process (Han et al., 2014; Moran, 2017). To the best of our knowledge, this is also the first study that provides some comparative evidence of flourishing and well-being in Sri Lanka and Cambodia. Finally, this study is not based on college students – perhaps the most frequent respondent group examined in the instrument validation studies.

### Operationalization of Flourish Index and Secure Flourish Index

Numerous composite measures of psychological well-being have been proposed and validated (Ryff and Keyes, 1995; Diener et al., 2010; Huppert and So, 2013; Su et al., 2014). It might even be argued that they should adequately measure well-being. However, as indicated above, they usually do not include character and virtue, and almost never – physical health. Therefore, two summary measures were devised (VanderWeele, 2017b). The first measure – the Flourish Index (FI) – included questions related to the first five domains enumerated above. The second measure – the Secure Flourish Index (SFI) – supplemented these five domains by adding questions on material and financial resources as supporting evidence for stability. The first index is perhaps more coherent and satisfactory as a conceptual measure of flourishing at any given time, as each domain inarguably signals its own end. In practice, however, the second measure may be superior. With recourse to financial and material sentiments, it is more likely to offer a better assessment of the conditions required for a sustained flourishing state over time.

For the proposed measures, two questions for each of the five (FI), or six (SFI), domains were suggested (VanderWeele, 2017b). The choice of questions and statements, hereafter called items, was based on frequency of their use in the literature in similar context and their empirical validation (Fordyce, 1988; Prawitz et al., 2006; OECD, 2013; Allin and Hand, 2017; NORC, 2017; WHO, 2017). The aim was to make use, whenever possible, of items that are already used in surveys, polls and other studies in order to benefit from prior empirical validation and facilitate comparisons – the approach that was also adopted by the United Kingdom Office for National Statistics in designing the ‘Measuring national well-being (MNW) program’ (Allin and Hand, 2017).

Consequently, the questions on life satisfaction, happiness, mental and physical health, and meaningful activities were selected as those already used by the Organisation for Economic Cooperation and Development (OECD, 2013), the United Kingdom Office for National Statistics (Allin and Hand, 2017), US General Survey (NORC, 2017), the World Mental Health Composite International Diagnostic Interview (WHO, 2017), and many others. The questions on close social relationships were taken from the Campaign to End Loneliness (2016) and the question about financial stability from Prawitz et al. (2006).

Two novel character and virtue questions were proposed because, although several scales for measuring specific virtues had been developed (Park et al., 2004; Peterson and Seligman, 2004), not many global single-item character and virtue questions available in the literature. Also, the question about material stability resulted from a modification of the question about financial stability, which originated from Prawitz et al. (2006).

Our measures – FI and SFI – refer to how people evaluate their lives and, in this sense, many of the items reflect eudaimonic sentiments. However, it is worth mentioning that in the life satisfaction and happiness domain (D1), there is a question about feeling happy (D1.2). This classic question of hedonic sentiment, due to longer temporal reference period (i.e., ‘In general, how happy or unhappy do you usually feel?’), also has somewhat of an evaluative character (National Research Council, 2013). Consequently, the life satisfaction and happiness domain (D1) captures judgments of overall life evaluations and refers specifically to life satisfaction and evaluative happiness, two concepts – as shown by Huppert and So (2013) – potentially measuring the same construct.

The mental and physical health domain (D2) does explicitly distinguish between physical (D2.1) and mental (D2.2) assessments. Although elements of the latter are included in psychological well-being, subjective well-being and flourishing measures in particular, assessment of the former is often disregarded. If used, physical health is applied as an outcome of psychological well-being (Hernandez et al., 2018) or subjective well-being (Diener et al., 2017) or as a correlate of flourishing (Schotanus-Dijkstra et al., 2015), three concepts, which do not focus on physical aspects of life. We argue, however, that if an overall assessment of complete well-being, or flourishing is of primary interest, then health – including physical health – should be included.

The meaning and purpose domain (D3) is the most classic eudaimonic measure of well-being and reflects the subjective value of one’s life (National Research Council, 2013). We explicitly distinguish between meaning of life (item D3.1) and purpose in life (item D3.2), which, despite often being used interchangeably, are in fact distinct constructs. Meaning reflects more of the existential dimension and refers to overall relatedness, coherence and significance of one’s experiences, whereas purpose mainly refers to pursuit and aspiration of certain ends, implying that it is more of a goal-oriented concept (King et al., 2006; George and Park, 2013).

The character and virtue domain (D4) is designed accordingly with the Aristotle’s (2009) conviction that in order to attain complete eudaimonic well-being, an excellent character and right manner behavior, understood as acting in accord with virtue, are essential. These theoretical considerations of Aristotle have been empirically examined with research showing that the exercise of character strengths does indeed contribute, on average, to increased human thriving and decreased depressive symptoms, with results lasting at least 6 months (Seligman et al., 2005). Consequently, out of the two character and virtue questions, the first (D4.1) is intended to provide some assessment of prudence and justice, and the second (D4.2), to reflect fortitude and temperance – four cardinal virtues of Plato and present also in Aristotle.

Close social relationships domain (D5) is intended to focus on quantity and quality of social connections both required and experienced. Similarly to the cognitive discrepancy theory of loneliness (Peplau and Perlman, 1979; Perlman and Peplau, 1981), we recognize that there is a discrepancy between the number and quality of relationships that one has and desires to have. However, in our approach, we focus on relationships instead of on loneliness, which refers to a social deficiency (Campaign to End Loneliness, 2016) and emphasize the gap between needed and experienced quantity and quality of social contacts (Peplau and Perlman, 1979; Perlman and Peplau, 1981). Therefore, out of two items in this domain, the first assesses the social connections experienced (D5.1), while the second (D5.2) evaluates fulfillment of the connections needed.

The financial and material stability domain (D6) is intended to take into account sustainability of flourishing and chances of preservation of five aforementioned aspects (operationalized as the domains) of human flourishing. The intention was to underscore that one should not think about flourishing as a momentary state only (VanderWeele, 2017b) but additionally focus on its preservation or enhancement. To this end, sufficiently stable both financial and material resources should be ensured. Since the distinction between importance of financial (called also income) and material resources for financial well-being has been confirmed (Ravallion, 2011; Alkire and Santos, 2013; Annoni and Wȩziak-Białowolska, 2016) we also distinguish between them asking distinctly about worrying about being able to meet normal monthly living expenses (D6.1) and worrying about safety, food, or housing (D6.2).

We are well aware that measurement of the above domains of well-being is complex, indirect and usually conducted using multi-item scales (Diener, 1984; Fordyce, 1988; Ryff and Keyes, 1995; Peterson and Seligman, 2004; Prawitz et al., 2006). Consequently, one might argue that if a new scale is developed, it should broaden the human flourishing concept by expanding not only its dimensions but also by raising the number of questions per dimension. While we generally agree with this argument, we also argue that long instruments – despite the advantages of conceptual richness – are sometimes less preferable to short instruments when used in the studies where flourishing is just one of many concepts being measured^1^. Despite some criticism of short instruments – especially those with one item per domain (Credé et al., 2012), which, when used, may lead to increased Type 1 and Type 2 error rates (see Credé et al., 2012 for evidence in the personality studies), these type of instruments can be found in psychology (Jonason and Webster, 2010; Maples et al., 2014), educational psychology (Ugen et al., 2014) and organizational behavior (Liden et al., 2015), among others. In the well-being field, short indices were proposed by Huppert and So (2013) whose flourishing index consists of 10 domains, called features, with only one item per domain. Additionally, the United Kingdom Office for National Statistics since 2011 asks a set of four well-being questions (only) in the UK National Survey (Allin and Hand, 2017). The aim was to keep the number of questions very limited to avoid excessive costs and to enable widespread use, while still allowing two items per domain.

## Materials and Methods

### Measures

#### Flourishing Index (FI)

Ten questions and statements – two per domain – belong to the Flourish Index set (Table 1).

**Table 1:** Flourish Index (FI) and Secure Flourish Index (SFI) – structure and items.

Measure	Domain	Statement/question
FI SFI	D1. Happiness and Life Satisfaction	D1.1 Overall, how satisfied are you with life as a whole these days?
		*0 = Not Satisfied at All, 10 = Completely Satisfied*
FI SFI	D1. Happiness and Life Satisfaction	D1.2 In general, how happy or unhappy do you usually feel?
		*0 = Extreme Unhappy, 10 = Extremely Happy*
FI SFI	D2. Mental and Physical Health	D2.1 In general, how would you rate your physical health?
		*0 = Poor, 10 = Excellent*
FI SFI	D2. Mental and Physical Health	D2.2 How would you rate your overall mental health?
		*0 = Poor, 10 = Excellent*
FI SFI	D3. Meaning and Purpose	D3.1 Overall, to what extent do you feel the things you do in your life are worthwhile?
		*0 = Not at All Worthwhile, 10 = Completely Worthwhile*
FI SFI	D3. Meaning and Purpose	D3.4 I understand my purpose in life
		*0 = Strongly Disagree, 10 = Strongly Agree*
FI SFI	D4. Character and Virtue	D4.1 I always act to promote good in all circumstances, even in difficult and challenging situations
		*0 = Not True of Me, 10 = Completely True of Me*
FI SFI	D4. Character and Virtue	D4.2 I am always able to give up some happiness now for greater happiness later
		*0 = Not True of Me, 10 = Completely True of Me*
FI SFI	D5. Close Social Relationships	D5.1 I am content with my friendships and relationships
		*0 = Strongly Disagree, 10 = Strongly Agree*
FI SFI	D5. Close Social Relationships	D5.2 My relationships are as satisfying as I would want them to be
		*0 = Strongly Disagree, 10 = Strongly Agree*
SFI	D6. Financial and Material Stability	D6.1 How often do you worry about being able to meet normal monthly living expenses?
		*0 = Worry All the Time, 10 = Do Not Ever Worry*,
SFI	D6. Financial and Material Stability	D6.2 How often do you worry about safety, food, or housing?
		*0 = Worry All the Time, 10 = Do Not Ever Worry, 10*

Each item is measured on an 11-point scale (from 0 to 10) with extreme categories labeled and oriented toward higher scores indicating more favorable responses. Average scores for each pair of items in specific domains constitute domain specific indices. FI scores are arithmetically averaged domain specific indices with equal weighting. FI and domain specific indices can range from 0.0 (the lowest response category chosen for all items) to 10.0 (the highest response category for all items). High scores imply that people perceive themselves very positively in terms of human flourishing. This means that FI should assess human functioning in all the important domains predicating human flourishing. Additionally, FI allows assessment of performance across domains, i.e., in terms of (1) life and satisfaction, (2) physical and mental health, and (3) meaning and purpose, etc.

#### Secure Flourish Index (SFI)

Two additional items, material and financial stability, in an extra sixth domain were used to augment FI to assess sustainability of a flourishing state over time. Items referred to availability of financial and material prerequisites to maintain the state. Both items are measured on an 11-point scale (from 0 to 10) with extreme categories labeled and oriented higher for more favorable responses. SFI scores are also calculated as the arithmetic average of all six domains with equal weighting. The D6 domain-specific score ranges from 0.0 (the lowest response category) to 10.0 (the highest response category).

Since human flourishing measures were used in culturally and linguistically diverse populations, the questions were translated from English to specific languages by professional translators. Then, to ensure cross-cultural comparability, English speaking students of the Harvard T.H. Chan School of Public Health, as well as independent translators from the countries where research was conducted, translated the questions back into English and discussed the differences with the research team. Finally, members of the Community Advisory Board for each country – who were English-speaking citizens of the country where the research was planned and who had appropriate professional expertise in human well-being – revised the English and country specific versions of questions in terms of their appropriateness (understanding, perception) for the clothing industry worker population.

### Participants

Data collection took place between June 2017 and March 2018 and was part of the SHINE Well-Being Survey. A total of 8866 respondents participated in the study: 4083 from the US (June 2017), 1284 from Sri Lanka (August 2017), 587 from Cambodia, 412 from China (both December 2017), 2500 from Mexico (March 2018) (Table 2). Responses were collected via online survey using the Qualtrics platform (all countries except Cambodia) or the offline Qualtrics tablet app (all countries except the US). For the US sample, the survey was a part of a project to examine the effects of a broad impact financial incentive on individual, family and community well-being in North Carolina. In the remaining countries, the survey was part of a project to examine determinants for the well-being of garment factory workers in the global supply chains of an international brand. Surveyed factory workers did not differ substantially from the respective populations of workers in factories visited in terms of gender, age and job tenure. In each case participation was voluntary. Informed written consent was obtained from participants. All protocols for recruitment and participation were reviewed and approved by the Harvard Longwood Medical Area Institutional Review Board. Data are available from the first author upon request.

**Table 2:** Characteristics of participants.

Characteristic	US (*n* = 4083)	Sri Lanka (*n* = 1284)	China (*n* = 419)	Cambodia (*n* = 587)	Mexico (*n* = 2500)
Females (%)	36.7	57.7	71.2	86.5	46.9
Age – mean (SD)	46.4 (14.6)	30.6 (9.2)	34.6 (9.8)	24.6 (4.9)	33.1 (10.9)
Education (higher than secondary education) (%)	59.5	59.4	20.5	18.9	36.8
Having children under 18 years old who currently live with the respondent (%)	26.2	46.1	82.6	56.7	62.3
Married (%)	57.4	57.9	81.1	60.5	40.0
% of the total factory workforce surveyed	—	32.8	64.2	19.8	58.3

### Statistical Analysis

The FI and the SFI are conceptualized respectively, as comprised of five and six related domains. This implies that they are multi-faceted constructs and they were tested as such. First, the US sample was randomly split into two subsamples. Exploratory factor analysis was applied to the first random subsample, *n* factor solutions (*n* = 1, … ,5 for the FI and *n* = 1,…,6 for SFI) were examined with respect to correspondence to the theoretical grouping of the items into domains (as shown in Table 1). Adequacy of our data for EFA was examined through the Keiser-Meyer-Olkin (KMO) measure and Bartlett’s test of sphericity. Oblimin rotation was applied, since it is well-established in the literature that domains of flourishing are correlated (Keyes, 2005).

**FIGURE 1:**
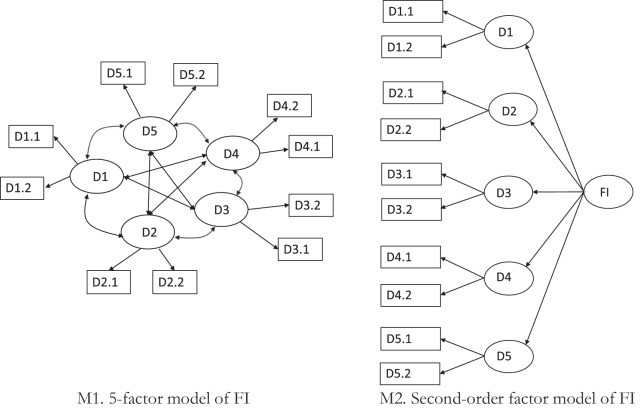
Two CFA specifications for the FI.

CFA was run on the second US subsample. Two specifications were tested. First, to examine whether item grouping was justified by the data, 5 (for FI) or 6 (for SFI)-factor models were tested (M1) with (1) one latent factor corresponding to each domain, (2) each item loaded only by a latent factor corresponding to the domain of an item and not by other latent factors, (3) correlated latent factors and (4) uncorrelated error terms. Secondly, a second-order factor model (M2) with (1) one latent factor corresponding to each domain, (2) each item loaded only by a latent factor corresponding to the domain of an item and not by other latent factors and (3) domain specific factors loaded by the second-order factor corresponding to human flourishing or secure human flourishing, for FI and SFI respectively (Xu et al., 2016). Schematics of these two specifications tested are shown in Figure 1.

At the validation stage, reliability analysis (Cronbach’s alpha) and CFA were run to assess the psychometric properties. Measurement invariances (configural, metric, and scalar) of FI and SFI in four additional and culturally distinct samples were examined using multi-group CFA.

Fit of CFA models was examined according to the comparative-fit index (CFI), the Tucker-Lewis index (TLI), the root-mean-square error of approximation (RMSEA), and standardized root mean square residual (SRMR). For RMSEA and SRMR, values less than 0.08 indicate a satisfactorily low level of noise in the model (Browne and Cudeck, 1992), and below 0.05 indicate a very low level of noise (Hu and Bentler, 1999). For the CFI and TLI, values greater than 0.95 indicate a satisfactory fit, although values greater than 0.90 are also acceptable (Hu and Bentler, 1999; Marsh et al., 2013). To compare three different specifications, fit indices as well as information criteria (AIC, BIC and sample-size adjusted BIC) were applied.

In tests of measurement invariance, the fit of the multi-group CFA was examined and differences in fit statistics between less and more restrictive models (configural measurement invariance versus metric measurement invariance versus scalar invariance) were analyzed (Putnick and Bornstein, 2016). Recommendations for large samples (*n* > 300) are that it is indicative of non-invariance when CFI changes less than -0.01 and RMSEA less than 0.015 (Chen, 2007). In the absence of full measurement invariance confirmation, partial measurement invariance (Steenkamp and Baumgartner, 1998; Putnick and Bornstein, 2016) was also examined.

CFA analysis was conducted using Mplus 8. Descriptive statistics and EFA were computed with Stata 15.

## Results

Table 3 presents the correlation matrix of FI and SFI items. Positive correlations were recorded between all items. We examined whether items within a specific domain correlated better than items between domains. This was confirmed for items from the last three domains (Character and Virtue, Close Social Relationships and Financial and Material Stability). Dominance of life satisfaction (D1.1) and happiness (D1.2) questions was clear for the remaining three domains.

**Table 3:** Flourish Index and SFI correlation matrix (US subsample 1; pairwise correlations).

Domain	Question/statement	D1.1	D1.2	D2.1	D2.2	D3.1	D3.2	D4.1	D4.2	D.5.1	D5.2	D6.1	D6.2
D1. Happiness and Life Satisfaction	D1.1 Overall, how satisfied are you with life as a whole these days?	**1**											
	D1.2 In general, how happy or unhappy do you usually feel?	**0.75**	**1**										
D2. Mental and Physical Health	D2.1 In general, how would you rate your physical health?	0.50	0.46	**1**									
	D2.2 How would you rate your overall mental health?	0.58	0.65	**0.49**	**1**								
D3. Meaning and Purpose	D3.1 Overall, to what extent do you feel the things you do in your life are worthwhile?	0.72	0.71	0.45	0.57	**1**							
	D3.2 I understand my purpose in life	0.55	0.64	0.36	0.56	**0.65**	**1**						
D4. Character and Virtue	D4.1 I always act to promote good in all circumstances, even in difficult and challenging situations	0.29	0.39	0.27	0.35	0.38	0.42	**1**					
	D4.2 I am always able to give up some happiness now for greater happiness later	0.27	0.35	0.23	0.31	0.33	0.36	**0.52**	**1.00**				
D5. Close Social Relationships	D5.1 I am content with my friendships and relationships	0.56	0.63	0.35	0.50	0.54	0.52	0.37	0.37	**1.00**			
	D5.2 My relationships are as satisfying as I would want them to be	0.58	0.63	0.35	0.51	0.56	0.51	0.38	0.36	**0.81**	**1.00**		
D6. Financial and Material Stability	D6.1 How often do you worry about being able to meet normal monthly living expenses?	0.33	0.26	0.25	0.21	0.24	0.17	0.05	0.10	0.19	0.19	**1.00**	
	D6.2 How often do you worry about safety, food, or housing?	0.31	0.29	0.24	0.25	0.25	0.18	0.05	0.06	0.22	0.21	**0.67**	**1.00**

*Correlations between the items from specific domain depicted in bold.*

Adequacy of our data for EFA was confirmed by KMO exceeding 0.8 and a significant chi-square value from Bartlett’s sphericity test (for both FI and SFI). Using EFA (Tables 4, 5, only US subsample 1), question grouping was found to support the theoretical grouping. Only questions in the Happiness and Life Satisfaction and the Meaning and Purpose domains were exceptions. Regardless of the dimensionality of the solution, they were always loaded by the same factor, perhaps suggesting a mutual cause. Additionally, EFA conducted on domain specific indices confirmed uni-dimensionality for FI and SFI.

**Table 4:** Factor loadings of exploratory factor analysis (US subsample 1) – FI.

Item	Two-factor solution		Three-factor solution			Four-factor solution				Five-factor solution				
	F1	F2	F1	F2	F3	F1	F2	F3	F4	F1	F2	F3	F4	F5
D1.1	0.818		0.866			0.608				0.745				
D1.2	0.786		0.767			0.635				0.585				
D2.1	0.634		0.615					0.553			0.593			
D2.2	0.727		0.685					0.413			0.618			
D3.1	0.811		0.772			0.794				0.856				
D3.2	0.679		0.579			0.719				0.561				
D4.1	0.305				0.632				0.616				0.634	
D4.2					0.616				0.601				0.627	
D5.1		0.943		0.921			0.946					0.942		
D5.2		0.969		0.957			0.982					0.972		

*KMO = 0.8703; Bartlett’s test of sphericity: χ^2^ = 21838.85; p-value = 0.000; Blanks are abs(loading) < 0.3. Description of questions is presented in Table 1.*

**Table 5:** Factor loadings of exploratory factor analysis (US subsample 1) – Secure Flourish Index.

Item	Two-factor solution		Three-factor solution			Four-factor solution				Five-factor solution					Six-factor solution					
	F1	F2	F1	F2	F3	F1	F2	F3	F4	F1	F2	F3	F4	F5	F1	F2	F3	F4	F5	F6
D1.1	0.675		0.737			0.861				0.619					0.723					
D1.2	0.784		0.783			0.799				0.637					0.559					
D2.1	0.449		0.581			0.561						0.537				0.576				
D2.2	0.652		0.717			0.691						0.424				0.596				
D3.1	0.734		0.808			0.811				0.806					0.848					
D3.2	0.723		0.745			0.639				0.716					0.567					
D4.1	0.586		0.503					0.625					0.617					0.628		
D4.2	0.539		0.422					0.621					0.611					0.629		
D5.1	0.888			0.925			0.917				0.946						0.943			
D5.2	0.869			0.958			0.955				0.985						0.977			
D6.1		0.726			0.714				0.757					0.765					0.761	
D6.2		0.721			0.712				0.743					0.752					0.754	

*KMO = 0.8355; Bartlett’s test of sphericity: χ^2^ = 24722.62; p-value = 0.000; Blanks are abs(loading) < 0.3. Description of questions is presented in Table 1.*

In the following step, 5 (for the FI) and 6 (for the SFI) factor models (M1) and the second-order factor model (M2)– as presented in Figure 1 – were estimated on the second US subsample. They were also estimated for other country specific samples.

The fit of models M1 and M2 was excellent with respect to both FI and SFI (Table 6), with minor reservations about the Chinese sample. The satisfactory fit of model M1 suggests that, first, the FI and SFI both have hierarchical structures, i.e., they are composed of items grouped according to their respective domains. Secondly, the domain specific indices are correlated yet distinct and could be aggregated into a composite measure, i.e., a domain specific index. Further, the satisfactory fit of the M2 model indicates that items of FI and items of SFI can be aggregated into a composite measure, i.e., the Flourish and Secure Flourish Indices, respectively.

**Table 6:** Confirmatory factor analysis – model fit.

Model	CFI	TLI	RMSEA	SRMR	χ^2^(*p*-value)	AIC	BIC	Sample-size adjusted BIC
*Flourish Index – M1 (5-factor model)*								
US sample 2	0.978	0.961	0.052	0.020	281.3 (0.000)	131958.6	132208.9	132081.8
Sri Lanka	0.968	0.943	0.047	0.030	96.7 (0.000)	55775.3	55981.5	55854.4
China	0.922	0.859	0.076	0.049	85.3 (0.000)	14633.0	14793.9	14667.0
Cambodia	0.933	0.988	0.021	0.024	31.3 (0.180)	19586.4	19761.4	19634.4
Mexico	0.982	0.967	0.028	0.021	74.0 (0.000)	92197.3	92430.1	92303.0
*Flourish Index – M2 (second-order factor model)*								
US sample 2	0.970	0.954	0.056	0.028	391.4 (0.000)	132131.5	13235.5	132239.2
Sri Lanka	0.949	0.924	0.055	0.039	144.7 (0.000)	55843.0	56023.4	55912.2
China	0.901	0.852	0.078	0.051	106.1 (0.000)	14658.8	14799.6	14688.5
Cambodia	0.983	0.975	0.030	0.032	46.1 (0.031)	19610.4	19763.6	19652.4
Mexico	0.955	0.933	0.04	0.036	150.0 (0.000)	92369.3	92573.0	92461.8
*Secure Flourish Index – M1 (6 factor model)*								
US sample 2	0.976	0.960	0.048	0.023	391.1 (0.000)	166105.9	166425.0	166262.9
Sri Lanka	0.968	0.946	0.043	0.028	131.1 (0.000)	68034.8	68297.6	68135.6
China	0.933	0.887	0.067	0.044	111.7 (0.000)	18781.4	18986.5	18824.6
Cambodia	0.994	0.989	0.020	0.024	47.8 (0.159)	25348.9	25572.1	25410.2
Mexico	0.985	0.974	0.025	0.019	102.2 (0.000)	115749.6	116046.6	115884.5
*Secure Flourish Index – M2 (second-order factor model)*								
US sample 2	0.965	0.952	0.053	0.034	568.0 (0.000)	166348.1	166610.9	166477.4
Sri Lanka	0.949	0.93	0.049	0.039	193.9 (0.000)	68112.9	68329.3	68195.9
China	0.914	0.882	0.069	0.047	141.2 (0.000)	18804.9	18973.8	18840.5
Cambodia	0.973	0.963	0.037	0.038	86.0 (0.000)	25397.3	25581.0	25447.7
Mexico	0.960	0.946	0.037	0.033	212.9 (0.000)	115934.0	116178.6	116045.1

The question arises whether FI and SFI and their domain specific scores are comparable in culturally distinct settings. To this end, the fit of multi-group CFA with imposed measurement invariance conditions was examined and the change in fit statistics analyzed (Table 7). Based on the fit statistics and the information criteria model, M1 had the best fit and measurement invariance was examined for this model.

**Table 7:** Multi-group confirmatory factor analysis – measurement invariance tests for model M1.

						
Model	CFI	ΔCFI < −0.01	TLI	ΔTLI	RMSEA	ΔRMSEA <0.015
*Flourish Index*						
Configural	0.974	–	0.954	–	0.042	–
Metric	0.968	−0.006	0.950	−0.004	0.044	0.002
Scalar	0.939	−0.029	0.917	−0.033	0.057	0.013
*Secure Flourish Index*						
Configural	0.975	–	0.957	–	0.040	–
Metric	0.970	−0.005	0.954	−0.003	0.041	0.001
Scalar	0.945	−0.025	0.925	−0.029	0.053	0.012

With respect to M1 specification, the fit of the configural model was excellent, which indicated that the factor structure of both indices is consistent between countries. This further implies that domains for (secure) flourishing appear to manifest a shared common understanding in the populations under study.

The maximum differences between fit statistics – CFI, TLI and RMSEA – while comparing a more restrictive metric model to a less restrictive configural model, were 0.006 for FI and 0.005 for SFI. This suggests that both the FI and SFI domain specific scores are metric invariant for all analyzed populations^2^. They are not, however, scalar invariant. Therefore, scalar partial measurement invariance was tested. When intercepts for Meaning and Purpose, Character and Virtue and Physical and Mental Health items were released, it sufficed to confirm sufficient fit of scalar invariant model (FI: CFI = 0.963; ΔCFI = 0.005; TLI = 0.945; ΔTLI = 0.005; RMSEA = 0.046; ΔRMSEA = 0.002; SFI: CFI = 0.964; ΔCFI = 0.006; TLI = 0.948; ΔTLI = 0.006; RMSEA = 0.044; ΔRMSEA = 0.003)^3^. This indicates that although comparing domain specific score means between analyzed populations is justified, some caution is appropriate with respect to items to assess Meaning and Purpose, Character and Virtue, Physical and Mental Health domains. Their unconstrained intercepts in the scalar invariant model may signify that respondents in some countries tended to agree (or disagree) consistently more with the respective items but this more marked agreement does not relate to increased Meaning and Purpose, Character and Virtue or Physical and Mental Health scores in these populations.

Table 8 presents means for the domain specific indices for each of the five study locations (according to the M1 model). Since our samples were not comparable in terms of basic demographics, the reported means were computed after weighting the non-US samples in terms of gender and age (grouped below 30, 30–39, 40–49, 50–59, 60, and above) to make them comparable to the US sample. We recognized that this approach might not be considered best practice but this was the best achievable approach to make comparisons between samples. We are aware that this might have influenced results and final conclusions (for not weighted results, please see Supplementary Table S1).

**Table 8:** Difference in mean domain specific scores (US sample 2 = reference group; *p*-values in parentheses; unstandardized estimates).

Domain	US	Sri Lanka	China	Cambodia	Mexico
D1. Happiness and Life	Ref.	−1.299^∗∗∗^	1.286^∗∗∗^	1.503^∗∗∗^	1.054^∗∗∗^
Satisfaction		(0.000)	(0.000)	(0.000)	(0.000)
D2. Physical and Mental	Ref.	0.036	1.896^∗∗∗^	1.580^∗∗∗^	1.595^∗∗∗^
Health		(0.805)	(0.000)	(0.000)	(0.000)
D3. Meaning and Purpose	Ref.	−0.303	1.114^∗∗∗^	1.446^∗∗∗^	1.175^∗∗∗^
		(0.204)	(0.000)	(0.000)	(0.000)
D4. Character and Virtue	Ref.	0.072	1.115^∗∗∗^	1.549^∗∗∗^	0.773^∗^
		(0.664)	(0.000)	(0.000)	(0.013)
D5. Close Social	Ref.	0.016	2.023^∗∗∗^	2.102^∗∗∗^	1.827^∗∗∗^
Relationships		(0.918)	(0.000)	(0.000)	(0.000)
D6. Financial and Material	Ref.	−0.732^∗∗∗^	−0.020	−1.712^∗∗^	−2.896^∗∗∗^
Stability		(0.000)	(0.929)	(0.001)	(0.000)

*^∗∗∗^p < 0.001, ^∗∗^p < 0.01, ^∗^p < 0.05; presented estimates are from SFI model M1; Estimates from FI model – not presented here – are very similar; available from the first author.*

US respondents scored the lowest in all domains of human flourishing with the exceptions of happiness and life satisfaction and financial and material stability, for which they obtained the highest score. Respondents in China scored highest in health domains. Life satisfaction and happiness, close social relationships in addition to character and virtue scores were highest in Cambodia. Respondents in Mexico obtained the lowest scores in financial and material stability, 2.9 points lower than the highest scoring American respondents. Respondents in Sri Lanka were the least happy or satisfied with life. Their scores in the domains of physical and mental health (D2), meaning and purpose (D3), character and strength (D4), and close social relationships (D5) were also the lowest and not significantly different from the scores of the American respondents. This was quite a surprising finding.

Correlations between domain specific indices across analyzed populations are presented in Supplementary Table S2. Cronbach’s alpha coefficients (Table 9) indicate excellent reliability of the FI and satisfactory reliability of the SFI in all populations analyzed.

**Table 9:** Reliability coefficients – Cronbach’s alpha – in all analyzed populations.

	US	Sri Lanka	China	Cambodia	Mexico
FI	0.905	0.846	0.879	0.895	0.822
SFI	0.875	0.806	0.811	0.816	0.763

## Discussion

This paper responded to the need for research on the validity and reliability of the Flourish and the Secure Flourish Indices articulated by VanderWeele (2017b). More specifically, we examined whether the two sets of questions to measure flourishing and secure flourishing can be used in empirical analysis as a composite score of (secure) flourishing and its domains.

Results confirmed the complex nature of both indices. In particular, analysis of the correlation matrix and EFA provided supporting evidence for grouping of the questions as reflected by the domains. Confirmatory factor analysis corroborated the complex structure of both indices. Reliability measures further confirmed satisfactory psychometric properties of both indices and supported use of factor scores for application to these approaches. Finally, establishment of configural, metric, and partial scalar measurement invariance evidenced both indices as culturally universal.

Our findings uniquely contribute to a clearer understanding of human flourishing and its geography. The analysis was enriched by the participation of two largely unexplored populations in Cambodia and Sri Lanka.

It is worth noting that prior to this study, Cambodia and Sri Lanka had never participated in any international comparative studies such as the World Value Survey or the International Social Survey Programme. This implies little knowledge about values, well-being and human flourishing in these populations. The only exceptions are the WHO’s Demographic and Health Survey (DHS) and the Gallup World Poll. While the former does not comprise abstract questions such as questions about life satisfaction, happiness, relationship with friends, general health, the Gallup World Poll is not freely available and does not publish raw data.

It must be noted, however, that despite surveying culturally diverse populations, our samples were not perfectly comparable in terms of basic demographics (gender, age, and educational level). Nevertheless, participants from Cambodia, China, Mexico and Sri Lanka all worked in the supply chain for the same global clothing brand, which better substantiated the basis for their comparison. However, to account for differences in sample compositions compared to the US sample, it should be remembered that to circumvent the imperfect comparability of samples, non-US samples were weighted with respect to gender and age.

According to the Flourish Index, the Cambodian and Chinese participants were the happiest and most satisfied with their life, followed by the Mexican, American, and Sri Lankan participants. The higher score of the Mexican participants compared, in particular, with that of the American participants, corroborating findings from the World Value Survey (Inglehart et al., 2014), in which Mexicans scored 8.5 and Americans – 7.4 (on a 1–10 scale when asked about satisfaction with life). Additionally, when asked about feeling happy, 94% of Mexicans agreed with the statement compared to 90% of Americans. Our findings are also in line with the Gallup Happiness Index, according to which 84% Mexican people declared themselves happy or very happy, compared to 64% of Americans (Gallup International, 2017).

In the Meaning and Purpose domain, the Cambodian participants were the highest scoring populations, followed by the Mexican and Chinese participants. Sri Lankan and American participants obtained the worst scores. When compared with the Global Purpose Well-being Index by Gallup, our results were rather discordant; 35% of Mexicans, 30% of Americans, 25% of Sri Lankans, 19% of Cambodians, and 11% of the Chinese thrived in terms of purpose. However, it is worth noting that the Global Purpose Well-being Index (Gallup World Poll, 2017) measures what respondents enjoy doing every day and their motivation to achieve personal goals – a more momentary and different operationalization to the one chosen in our study.

Our results clearly highlighted cultural differences in Happiness and Life Satisfaction and Meaning and Purpose domains. Respondents from interdependent cultures usually scored similarly in contrast to the American respondents. However, our results indicated that participants from an individualistic culture, i.e., Americans, scored lower in both domains than participants from interdependent cultures. These results are at odds with what was reported by Steger et al. (2008) and Kitayama et al. (2010) for Americans and the Japanese. The differences that we observed may result from: (1) lack of a Japanese sample in our study, (2) the fact that in the Steger et al. (2008) study, respondents were undergraduate psychology students, which substantially limited generalizability of their findings. Our research is however consistent with the conclusion that a meaning to life is strongly related to higher well-being (see Supplementary Table S2), as reported by Steger et al. (2008) and Kitayama et al. (2010).

We related our results in the Character and Virtue domain to two questions from the World Value Survey (Inglehart et al., 2014). When respondents were asked whether most people, given the chance, would attempt to take advantage of them or try to be fair, people in China scored 6.9, in Mexico – 6.1 and in the US – 5.7. The 1 to 10 scale was: 1-they would try to take advantage; 10-they would try to be fair. Additionally, when requested to compare their own behavior and attitude in response to the statement, “It is important to a person to always behave properly; to avoid doing anything people would say is wrong,” 59.9% of respondents in Mexico responded “Like me” or “Very much like me” compared with 37.8% in the US and 34.1% in China. Our findings complemented findings from previous studies. According to the Character and Virtue domain of the human flourishing, the highest scoring were the Cambodian respondents, followed by the Chinese respondents and Mexican participants, with the scores of the Sri Lankan and American participants being the lowest and not significantly different. Again, clear distinction was observed between collectivist and individualistic cultures. The lowest scores of the American respondents were in line with the winner-take-all mentality. The decisively highest scores of participants from Cambodia, China and Mexico, were as anticipated from beyond-the-self cultures, as suggested by Greene (2013) and Moran (2017) for the American and Japanese and by Kim et al. (2012) for the American and South Korean college students. However, scores of the Sri Lankan participants were at odds.

In the Close Social Relationships domain, we found that respondents from Cambodia, China and Mexico scored the highest and respondents from Sri Lanka and the US – the lowest. This picture is not confirmed by the Gallup Global Social Well-being Index. According to this metric, Mexicans and Americans thrived best in terms of supportive relationships and love in life, while the Chinese scored the lowest. However, our results corresponded to the cultural background of participants (with exception of the Sri Lankan participants). High scores of participants with interdependent cultural backgrounds confirmed the values they attached to social harmony and perceived emotional support from close others (Kitayama et al., 2010). Low scores of the American respondents were, in turn, in line with the motivations of individualistic cultural norms which are focused on personal control and independence, thus depending less on social others (Kitayama et al., 2010).

The most perplexing findings of our study are in the Physical and Mental Health domain. Our results indicate that the Chinese participants, closely followed by Mexican and Cambodian respondents, most positively assessed their health, while the Sri Lankan and American respondents were the least positive. Objective health indicators published by the World Health Organization (WHO) suggested a rather different ranking. For example, healthy life expectancy at age 60 in 2015 amounted to 18.1 in the US, 17.1 in Mexico, 16.3 in Sri Lanka, 15.9 in China and 10.5 in Cambodia (WHO, 2018). Crude suicide rate in 2015 in Sri Lanka was 35.5, in the US it was 14.3, in Cambodia – 11.9, and in China – 10.0. It was the lowest in Mexico – 5.0 (WHO, 2018). It is apparent that while Mexicans are among the top performers on both indicators, the Chinese are average while Cambodians are among the lowest scorers. Additionally, when compared with percentages of positive assessment of general health, the picture appeared even more dissonant. According to the OECD Better Life Index (OECD, 2018), when asked about general health, 66% of people in Mexico and 88% of people in the United States reported good health. However, when asked about physical health, 73% of the Chinese, 65% of Mexicans, 62% of Sri Lankans, 59% of Americans, and 47% of Cambodians agreed with the statement “My physical health is almost perfect” (Gallup World Poll, 2017).

Following the reasoning of Wȩziak-Białowolska (2014), who identified substantial metric-dependent variation in health conditions in European regions, we hypothesize that the above discrepancies between objective and subjective health measures might not only be explained by inconsistency in the understanding of the concept of general health and assessment of health (Jylhä, 2009), but also by health ideals related to awareness (Graham, 2008; Filipkowski et al., 2010). These ideals and awareness are closely related to health care service provision and use-frequency (Murray and Chen, 1992), not discounting levels of health literacy (Kickbush et al., 2013). Puzzling American scores may stem from impact on health awareness and ideals of constant public debate concerning perceived deficiencies in the US health care system. The dissident unfavorable health assessment recorded in this study is also more explicable in terms of the repeal of the ‘Affordable Care Act’ of 2010, known as ‘Obamacare’ and in conjunction with selection of the US study sample. This was drawn from North Carolina, which ranked only 33^rd^ out of 50 states in 2017 in the America’s Health Rankings and was well below average (United Health Foundation, 2017). Additionally, Americans in the sample were considerably older (see Table 2) members of a credit union, while other samples were workers – younger and reasonably healthy to be able to work. Finally, our questions referred separately to physical and mental health; therefore, comparisons based on questions concerning general health should be accompanied with some caution.

Significant differences in the Financial and Material Stability domains between the American and other groups closely reflect previous research findings and official statistics. According to the International Monetary Fund^4^, GDP per capita expressed in international dollars, adjusted for purchasing power parity in 2017, amounted to 4010.2 in Cambodia, 13000.8 in Sri Lanka, 16624.4 in China, 19479.6 in Mexico, and 59495.3 in the US. These figures correlate well with scores in the Financial and Material Stability domains. Mexico, however, where scores using our measure were the lowest, is one exception to this pattern. Another one is China, in which our measure showed close similarities with the American respondents. To understand this issue better, we examined questions from the World Value Survey (Inglehart et al., 2014) and the Gallup World Poll (Gallup World Poll, 2017). When asked whether they worried about money, 63% of Mexican respondents agreed, compared with 44% of Sri Lankans, 43% of Cambodians, 39% of Americans, and 35% of Chinese (Gallup World Poll, 2017). When asked about satisfaction with the financial situation of their households, Mexican respondents scored 7.0 (1-completely dissatisfied, 10-completely satisfied) compared to scores of 6.2 from respondents both from China and the US (Inglehart et al., 2014). Additionally, when asked about how often the respondent or their family had gone without enough food to eat in the last 12 months, 18.1% people from Mexico reported at least sometimes, compared to 11.5% in US and 2.8% in China (Inglehart et al., 2014). Finally, when asked about feeling vulnerable to crime in their own homes, 13.8% people from Mexico reported often having such feelings and an additional 27.6% – sometimes; respective figures for respondents from the US were 1.6 and 9.6%, and for people in China – 0.7 and 4.8% (Inglehart et al., 2014). These findings highlight sensitivities to assessments according to choice of indicators. As pointed out by other scholars (Białowolski and Wȩziak-Białowolska, 2014; Annoni and Wȩziak-Białowolska, 2016; Tibesigwa et al., 2016), subjective and objective measures and also absolute and relative metrics for income, financial well-being and poverty, yield different results. Scrutiny of these deviations, however, may offer a deeper understanding of the phenomena. In this light, we contend that our findings in the Financial and Material Stability domain may only be superficially contentious.

## Conclusion

The measurement of human flourishing – the ability of humans to thrive – has potential to inform policy and personal reflection, to guide design of interventions and to monitor societal well-being. We believe that the Flourish Index and the Secure Flourish Index will be valuable tools for these objectives. Their psychometric properties recommend their suitability as measurement instruments. Offering unique evaluations for Sri Lanka and Cambodia, findings from this study enrich our knowledge about how humanity flourishes.

## Ethics Statement

This study was carried out in accordance with the recommendations of The Harvard LMA Schools’ Human Research Protection Program (HRPP), the Harvard Longwood Medical Area Institutional Review Board. The protocol was approved by the Harvard Longwood Medical Area Institutional Review Board. All subjects gave written informed consent in accordance with the Declaration of Helsinki.

## Author Contributions

DW-B designed the research, performed the research, analyzed the data, and wrote the manuscript. EM and TV designed the research and revised the manuscript.

## Conflict of Interest Statement

The authors declare that the research was conducted in the absence of any commercial or financial relationships that could be construed as a potential conflict of interest.

## Footnotes

1 One such study is on workers’ well-being in which well-being/flourishing is only one aspect of interest along with physical and psycho-social working conditions, work safety and occupational health, job burden, job autonomy, job resources, work-family conflict, and others. In such a situation, a less time-consuming instrument could be of a certain value.

2 Metric invariance means that a unit change in the domain of FI (or the SFI) has the same meaning in all populations. It further implies that the strength of association/relationship between domain of flourishing (secure flourishing) and other measures can be compared across populations. For example, assuming that the correlation coefficient for association between Meaning and Purpose score and job satisfaction equals 0.9 for the Chinese and 0.5 for the Americans. Only if metric invariance is ensured is the statement that *“meaning and purpose score is more correlated with job satisfaction among the American than among the Chinese”* valid.

3 Scalar invariance means that not only is the measurement unit of the FI (of the SFI) the same in all analyzed populations but also the regression intercepts of latent variables on indicator variables. Without scalar measurement invariance, differences in averages across populations could be an artifact of higher levels of zero (neutral) assessments in one of the populations, i.e., they may stem from higher or lower intrinsic optimism/pessimism or response styles, to name just a few factors, in a given subpopulation.

4 http://www.imf.org/en/Data
