# Supplementary material for: Human Flourishing in Cross Cultural Settings. Evidence From the United States, China, Sri Lanka, Cambodia, and Mexico
Source: Front Psychol. 2019 May 29;10:1269. doi: 10.3389/fpsyg.2019.01269 (PMC6549439; doi:10.3389/fpsyg.2019.01269)
Supplement: Supplementary file 1 [file Table_1.docx]

Table S1. Difference in mean domain specific scores (US sample 2 = reference group; p-values in parentheses; unstandardized and not weighted estimates)

| Domain | US | Sri Lanka | China | Cambodia | Mexico |
| --- | --- | --- | --- | --- | --- |
| D1. Happiness and Life Satisfaction | Ref. | -0.969***  (0.000) | 0.885***  (0.000) | 1.110***  (0.000) | 1.087***  (0.000) |
| D2. Physical and Mental Health | Ref. | 0.396***  (0.000) | 1.626***  (0.000) | 1.014***  (0.000) | 1.533***  (0.000) |
| D3. Meaning and Purpose | Ref. | 0.045  (0.631) | 0.730***  (0.000) | 1.086***  (0.000) | 1.299***  (0.000) |
| D4. Character and Virtue | Ref. | 0.480***  (0.000) | 0.762***  (0.000) | 1.058***  (0.000) | 0.949***  (0.000) |
| D5. Close Social Relationships | Ref. | 0.392***  (0.000) | 1.800***  (0.000) | 1.702***  (0.000) | 1.686***  (0.000) |
| D6. Financial and Material Stability | Ref. | -0.899***  (0.000) | -0.633***  (0.000) | -2.388***  (0.000) | -3.656***  (0.000) |

****p<0.000; presented estimates are from SFI model; Estimates from FI model – not presented here - are very similar; available from the first author;*

Table S2. Correlation coefficients for domain specific indices for US, China, Cambodia, Sri Lanka and Mexico.

|  | D1 | D2 | D3 | D4 | D5 | D6 |
| --- | --- | --- | --- | --- | --- | --- |
| US |  |  |  |  |  |  |
| D1. Happiness and Life Satisfaction | 1 |  |  |  |  |  |
| D2. Physical and Mental Health | 0.892*** | 1 |  |  |  |  |
| D3. Meaning and Purpose | 0.932*** | 0.830*** | 1 |  |  |  |
| D4. Character and Virtue | 0.625*** | 0.677*** | 0.701*** | 1 |  |  |
| D5. Close Social Relationships | 0.785*** | 0.715*** | 0.737*** | 0.641*** | 1 |  |
| D6. Financial and Material Stability | 0.333*** | 0.336*** | 0.286*** | 0.149*** | 0.215*** | 1 |
| China |  |  |  |  |  |  |
| D1. Happiness and Life Satisfaction | 1 |  |  |  |  |  |
| D2. Physical and Mental Health | 0.642*** | 1 |  |  |  |  |
| D3. Meaning and Purpose | 1.000^†^ | 0.759*** | 1 |  |  |  |
| D4. Character and Virtue | 0.589*** | 0.544*** | 0.724*** | 1 |  |  |
| D5. Close Social Relationships | 0.678*** | 0.682*** | 0.918*** | 0.584*** | 1 |  |
| D6. Financial and Material Stability | 0.180* | 0.036*** | 0.210** | 0.080** | 0.161* | 1 |
| Cambodia |  |  |  |  |  |  |
| D1. Happiness and Life Satisfaction | 1 |  |  |  |  |  |
| D2. Physical and Mental Health | 0.528*** | 1 |  |  |  |  |
| D3. Meaning and Purpose | 1.000^†^ | 0.601*** | 1 |  |  |  |
| D4. Character and Virtue | 0.845*** | 0.618*** | 0.921*** | 1 |  |  |
| D5. Close Social Relationships | 0.820*** | 0.579*** | 0.916*** | 0.767*** | 1 |  |
| D6. Financial and Material Stability | 0.083 | 0.325* | 0.063 | 0.055 | 0.013 | 1 |
| Sri Lanka |  |  |  |  |  |  |
| D1. Happiness and Life Satisfaction | 1 |  |  |  |  |  |
| D2. Physical and Mental Health | 0.675*** | 1 |  |  |  |  |
| D3. Meaning and Purpose | 0.918*** | 0.612*** | 1 |  |  |  |
| D4. Character and Virtue | 0.710*** | 0.586*** | 0.948*** | 1 |  |  |
| D5. Close Social Relationships | 0.642*** | 0.547*** | 0.636*** | 0.703*** | 1 |  |
| D6. Financial and Material Stability | 0.159*** | 0.068 | 0.016 | 0.107** | 0.150*** | 1 |
| Mexico |  |  |  |  |  |  |
| D1. Happiness and Life Satisfaction | 1 |  |  |  |  |  |
| D2. Physical and Mental Health | 0.722*** | 1 |  |  |  |  |
| D3. Meaning and Purpose | 1.000^†^ | 0.689*** | 1 |  |  |  |
| D4. Character and Virtue | 0.717*** | 0.841*** | 0.886*** | 1 |  |  |
| D5. Close Social Relationships | 0.711*** | 0.660*** | 0.544*** | 0.729*** | 1 |  |
| D6. Financial and Material Stability | 0.223** | 0.166 | 0.170 | 0.178 | 0.146 | 1 |

****p<0.000, **p<0.01, * p<0.05; ^†^ correlation fixed to 1 due to identification requirements;*
